# Effects of immediate and delayed nutrient timing following resistance exercise on changes in mixed muscle fractional synthesis rate (FSR) in post-menopausal women participating in a weight loss program

**DOI:** 10.1186/1550-2783-10-S1-P3

**Published:** 2013-12-06

**Authors:** M Byrd, S Simbo, YP Jung, B Sanchez, M Cho, CW Lee, B Lockard, C Baetge, K Levers, E Galvan, A Jagim, JM Oliver, R Dalton, B Bessire, K Horrell, T Leopold, M Koozehchian, D Khanna, K Shimkus, W Gapinsky, M Perez, J Hart, S Riechman, J Fluckey, M Greenwood, C Rasmussen, R Kreider

**Affiliations:** 1 Department of Health and Kinesiology, Exercise and Sport Nutrition Laboratory, Texas A&M University, College Station, TX 77843-4243, USA

## Background

Ingestion of protein prior to and/or following resistance-exercise (RE) has been reported to stimulate protein synthesis. Moreover, previous research from our lab found that older women who followed a higher protein hypo-energetic diet while participating in a RE program experienced more favorable changes in body composition than those following a higher carbohydrate diet. Theoretically, ingesting protein following RE during a weight loss program may stimulate protein synthesis to a greater degree, therefore helping to preserve and/or increase fat free mass (FFM). The purpose of this study was to investigate the effects of immediate vs. delayed post-exercise intake of a commercially available protein supplement on muscle protein fractional synthesis rate (FSR) prior to and following participation in a RE based exercise and weight loss program in post-menopausal overweight women.

## Methods

In a randomized and matched manner, 21 sedentary women (59.8±5 yr, 43.7±3% body fat, 31.0±3 kg/m^2^) participated in the Curves Complete^®^ weight loss and circuit resistance-exercise program for 12-wks. Participants followed an energy-restricted diet (1,500 kcal/d; 30% C, 45% P, and 25% F) while participating in a circuit resistance-training (3 d/wk) and walking (10k steps, 4/d wk) program. Participants ingested a drink containing 15 g of protein immediately following (I) or 2-hr after (D) resistance exercise as part of their diet program. DEXA, body composition and muscle FSR were determined prior to and following the exercise and diet intervention. A stable isotope Deuterium Oxide (D_2_O or ^2^H_2_O) ingestion methodology was utilized, and muscle biopsies obtained from the right (pre training) and left (post-training) vastus lateralis muscle in order to assess the effect of nutrient timing on mixed muscle FSR with, or without RE training. The advantage of this methodology is that FSR can be assessed over a 24 h period to determine the influence of exercise and/or nutrient timing on the total daily anabolic response. Data were analyzed by repeated measures MANOVA and ANOVA.

## Results

Participants in both groups lost weight (-3.9±3.2 kg, p=000) and fat mass (-4.1±2.4 kg, p=0.000) with no significant differences (mean±SD) observed among groups in weight (I -3.6±2.3; D -4.2±4.2 kg, p=0.68) or fat mass (I -3.5±1.4; D -4.8±3.3, p=0.26). FFM tended to increase (0.5±1.6 kg, p=0.12) with no differences observed among groups (I 0.03±1.7; D 1.11±1.3 kg, p=0.14). Based on prior analyses, no significant nutrient timing x training interactions (mean±SEM) were observed on muscle FSR expressed as a percent/day of the alanine pool (I-Pre 13.6±4.3, I-Post 21.1±4.3; D-Pre 15.6±4.0, D-Post 23.8±4.0 %/d, p=0.93). However, FSR was augmented (p<0.05) in response to a bout of RE prior to training (14.6±2.9 %/d) and tended to be 54% higher (p=0.075) in response to a bout of exercise after training when compared to pre-training values (22.5±2.9 %/d).

## Conclusions

Results indicate that the exercise and diet program investigated was effective in promoting weight and fat loss without loss in FFM. The exercise program was also effective in stimulating muscle protein synthesis prior to training. This stimulus persisted, and tended to be more pronounced following 12-wks of training. However, while some trends were observed warranting additional research, there did not appear to be any advantage of immediate or delayed nutrient timing on 24-h FSR in this population. These findings suggest that, rather than the timing of ingestion, daily nutrient intake may be the primary concern when it comes to maintaining muscle protein anabolism with exercise.

## Funding

Supported by Curves International, Waco, TX

